# Determinants of Diet and Physical Activity in Malaysian Adolescents: A Systematic Review

**DOI:** 10.3390/ijerph16040603

**Published:** 2019-02-19

**Authors:** Shooka Mohammadi, Muhammad Yazid Jalaludin, Tin Tin Su, Maznah Dahlui, Mohd Nahar Azmi Mohamed, Hazreen Abdul Majid

**Affiliations:** 1 Department of Social and Preventive Medicine, Centre for Population Health, Faculty of Medicine, University of Malaya, Kuala Lumpur 50603, Malaysia; shooka.mohammadi@um.edu.my (S.M.); tstin@ummc.edu.my (T.T.S.); maznahd@ummc.edu.my (M.D.); 2 Department of Paediatrics, Faculty of Medicine, University of Malaya, Kuala Lumpur 50603, Malaysia; yazidj@ummc.edu.my; 3 South East Asia Community Observatory (SEACO), Jeffrey Cheah School of Medicine and Health Sciences, Monash University Malaysia, Bandar Sunway 47500, Malaysia; 4 Faculty of Public Health, Universitas Airlangga, Jawa Timur 60115, Indonesia; 5 Department of Sports Medicine, Faculty of Medicine, University of Malaya, Kuala Lumpur 50603, Malaysia; nahar@ummc.edu.my; 6 Department of Nutrition, Harvard T.H. Chan School of Public Health, Harvard University, Boston, MA 02115, USA

**Keywords:** eating habits, Malaysian adolescents, physical activity, systematic review

## Abstract

The increased prevalence of unhealthy eating habits and sedentary lifestyles among Malaysian adolescents has become a public health concern. The aim of this systematic review was to summarize evidence from observational studies related to diet and physical activity (PA) among Malaysian adolescents (13–18 years) and to recognize the associations between determinants of diet and PA and diet and PA behaviours. A systematic search for observational studies published from August 1990 through August 2017 was conducted via PubMed, Science Direct, Cochrane and Web of Science. A total of 18 studies met the inclusion criteria; these were independently extracted by two reviewers. Gender and ethnicity were the most commonly studied correlates of diet and PA; males were more physically active and they tended to have poorer diet quality and higher energy and macronutrient intakes in comparison to females; Malay adolescents had a lower diet quality and Chinese adolescents spent less time in PA compared to other ethnicities. However, the significance of these associations was often small or inconsistent. This review highlights the lack of longitudinal observational studies but summarizes the best available evidence for policymakers and public health practitioners to improve the diet and the level of PA in Malaysian adolescents.

## 1. Introduction

The increasing prevalence of unhealthy eating habits and sedentary lifestyles among adolescents, which often continues into adulthood, is a global public health problem [1,2]. Increased intake of foods that are high in energy density, fat, and sugar and in combination with sedentary lifestyles (being physically inactive) and low levels of physical activity (PA) are major contributors to obesity in adolescents [3,4,5].

Negative longitudinal and secular trends have been reported in dietary behaviours among adolescents, such as an adverse change in dietary patterns and a decline in dietary quality during the transition from childhood to adolescence, and particularly decreases in fruit, vegetable, milk, and fruit juice consumption, and an increase in sugar-sweetened beverage (SSB) consumption [6,7]. Furthermore, portion sizes and frequency of fast food and snack intake have increased in the past decade [8,9]. There is an increasing trend of unhealthy eating among adolescents worldwide, which is contributing to an increase in the incidence of obesity [7] and the development of chronic diseases such as diabetes and cardiovascular disease [10,11].

It has been revealed that the eating patterns of children and adolescents are related to characteristics of both the social and physical environment [12]. They are eager to eat foods that are easily available and reachable, and they have a tendency to eat higher amounts when larger food portions are prepared. Moreover, socioeconomic and sociocultural factors such as parents’ educational level, ethnicity, time constraints, and mealtime structure including whether families eat together, the source of foods (e.g., schools, restaurants), and TV-viewing during meals are related to the eating patterns of children and adolescents [12]. In a recent review, feeding strategies and parental food habits were the most dominant determinants of a child’s eating behaviour and food choice [12].

In addition, gender has also been found to be associated with dietary behavior in adolescents [2]; girls have greater or more frequent consumption of fruits and vegetables compared to boys [13]. An association between obesity and low intakes of fruits and vegetables and overweightness has been reported among this age group [14]. There were also positive relationships noted between parental encouragement, family rules, home availability, and fruit and vegetable consumption [15].

Globally, physical inactivity and low fitness in children and adolescents are contributing to a rising health burden [16]. Several systematic reviews have reported that adolescents spend most of their time engaged in sedentary activities [17,18,19]. Regular participation in PA has been shown to produce significant health benefits for adolescents such as obesity prevention, improved psychological well-being, cardiovascular fitness and bone health [20,21]. Additionally, PA behaviours adopted during adolescence are likely to be maintained into adulthood [22,23]. The global trend shows that girls are less active than boys [24] and there is a greater reduction in PA during adolescence for girls compared to boys [25].

Despite the health benefits linked to PA, a recent literature review has revealed that PA levels decline across the lifespan and particularly during adolescence. This is in line with findings reported in Malaysian adolescents [26]. Inadequate PA and increased prevalence of obesity among Malaysian adolescents, especially for rural female adolescents, is a public health concern [27]. In Malaysia, there are 5.5 million adolescents aged 10–19, which equates to 18.9% of the total population [28]. The prevalence of unhealthy lifestyles as well as the proportion of overweight and obese Malaysian adolescents have both increased in recent decades [29]. Thus, the promotion of PA and healthy eating in this age group has become an increasingly important priority in order to promote health, prevent disease, and reduce the prevalence of obesity [30].

In recent years, several observational studies have been conducted to understand which factors are related to the dietary patterns and PA of Malaysian adolescents [27,31,32]. However, there seems to be a lack of well-conducted studies and the various methodological approaches that have been adopted thus far provide insufficient evidence-based information regarding the dietary patterns and PA of Malaysian adolescents. Therefore, the main aim of this systematic review was to summarize the evidence from observational studies related to diet and PA in Malaysian adolescents in secondary schools (13–18 years old). Another aim was to understand the determinants of diet and PA and their associations with these behaviours. Hopefully, the findings of this systematic review will be used to develop interventions that might improve the eating patterns and levels of PA, as well as reduce the sedentary behaviours among adolescents in Malaysia.

## 2. Methods

The Preferred Reporting Items for Systematic Reviews and Meta-Analyses (PRISMA) statement for systematic reviews was used to structure the present review and increase its integrity (Table S1).The review protocol was registered in the International Prospective Register of Systematic Reviews database (PROSPERO) (registration number: CRD42017074556).

### 2.1. Literature Search

One reviewer implemented the search strategy to search for relevant articles published from August 1990 up through August 2017 on the PubMed, Science Direct, Cochrane Review and Web of Science. Two reviewers then independently screened the identified studies against inclusion criteria and before extracting data. The search strategy was inclusive and concentrated on three key elements: population (e.g., Malaysian adolescents, secondary school), observation (e.g., diet and PA) and outcomes (e.g., associations with diet and PA behaviours) (Text S1).

### 2.2. Selection of Studies

This systematic review included all observational studies that looked at the determinants of diet and/or PA and their associations with these behaviours among Malaysian adolescents. The studies selected for analysis were required to meet the following inclusion criteria: (i) reported associations between diet and/or PA factors and diet and/or PA behaviours; (ii) involved healthy Malaysian adolescents aged between 13 and 18 years; (iii) were fully published articles. Studies were excluded based on the following criteria: (i) population studies that included children younger than 13 years of age or adults older than 18 years of age; (ii) papers that were not peer-reviewed or were only abstracts; (iii) studies that recruited adolescent subjects on the basis of disease, e.g., obese, diabetic, etc.; (iv) studies that did not report on the associations between diet/PA determinants and the diet/PA behaviour as the outcomes; (v) studies that included subjects that were not Malaysian.

In cases where the selected papers did not present all the necessary information, the corresponding authors were contacted to obtain further details in order to establish the eligibility of the paper for inclusion in this systematic review. To identify the appropriate studies, one reviewer (S.M.) reviewed all the titles and the abstracts retrieved through the database searches. Then, an initial screening of the title and the abstract was conducted by two reviewers (S.M. and Z.T.) against the above-mentioned exclusion criteria. Next, an evaluation of full-text articles was conducted by the review team (S.M. and Z.T.) to refine the results based on the inclusion criteria. Disagreements between the reviewers were resolved through discussion with L.J. and H.A.M. Among the 25 studies that were initially captured, seven studies were excluded because they reported mixed age ranges or some adolescents below 13 years old.

### 2.3. Quality Assessment

The quality of the studies was assessed using the modified Newcastle-Ottawa Scale for cohort and cross-sectional studies [33] (Text S2). It was developed to assess the quality of studies in three domains selection of study groups, comparability of groups and ascertainment of exposure and outcomes. Two reviewers (S.M. and Z.T.) did pilot testing of the quality assessment tools independently on four of the included studies, and then completed the evaluation of the quality of the remaining studies, independently. Where there were disagreements, the two reviewers discussed the issues until consensus was achieved. Studies were determined as being good, fair, or poor. “Good quality “comprised three or four stars in the selection domain, one or two stars in the comparability domain, and two or three stars in the outcome/exposure domain. “Fair quality” had two stars in the selection domain, and one or two stars in the comparability domain and two or three stars in the outcome/exposure domain. “Poor quality” had zero or one star in the selection domain, zero stars in the comparability domain, and zero, or one star in the outcome/exposure domain [33]. Tables S2 and S3 provide a summary of the quality assessment.

### 2.4. Data Extraction

Data was extracted by two independent reviewers (S.M. and Z.T.) into a standardized Excel spreadsheet (Microsoft Corporation, Albuquerque, NM, United States) and included a range of study characteristics like author, year of publication, study setting, study design, methodology, sample size, participants’demographics (e.g., age, ethnicity, maternal education, income), physical activity, dietary assessment, covariates, as well as the main exposures and outcomes. Data was also extracted on specific diet/PA factors, associations between the potential determinants of diet/PA and diet/PA behaviours, and characterization of outcomes (diet, PA). In addition, differences between reviewers were infrequent (concordance > 95%). Discrepancies were resolved through discussion by consensus.

### 2.5. Data Synthesis

Associations were considered significant when the *p*-value in the study was < 0.05. For those studies that performed both univariate and multivariate analyses, only the multivariate results were reported in this review. This was done because the results of multivariate analyses provide more accurate associations, and because they control for other potential confounding variables. Also, conceptually similar correlates and determinants of diet and PA were combined to reduce the number of variables considered in this review.

## 3. Results

**Figure 1:**
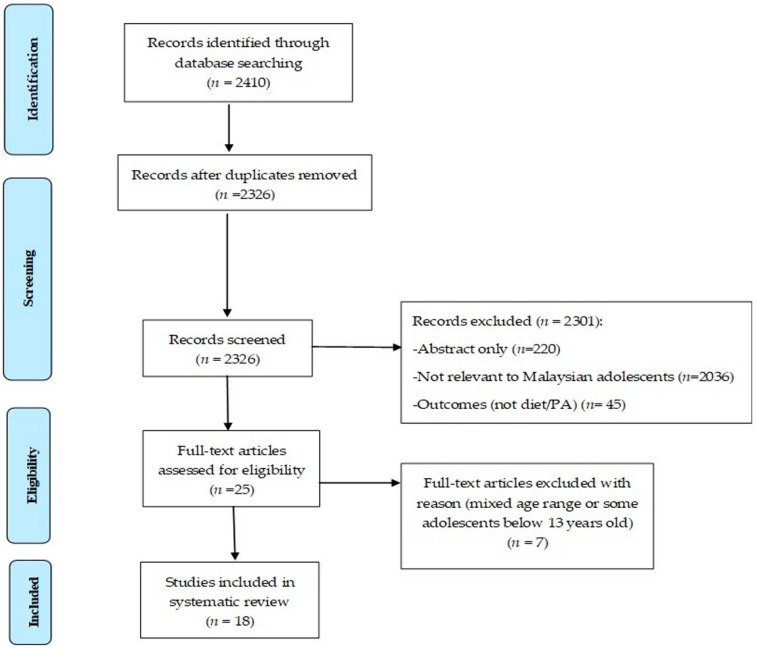
PRISMA Flow Diagram of study selection.

The initial multi-database search yielded 2410 publications. From this, 18 were included in this systematic review (Figure 1). The selected studies focused on associations between the determinants of diet and PA as well as their associations with diet and PA behaviours in Malaysian adolescents. Seventeen studies were cross-sectional [31,34,35,36,37,38,39,40,41,42,43,44,45,46,47,48,49]; only one was a prospective cohort study [27].

Table 1 provides a summary of each study. From the table, it can be seen that the sample sizes of the studies ranged from 156 to 40,011 adolescents. Seventeen studies included both boys and girls (*n* = 17); one study was conducted among girls [31]. The majority of the studies included adolescents from different ethnicities, i.e., Malay, Indian and Chinese (*n* = 12). Nearly half of the studies were conducted in central Malaysia and Kuala Lumpur (*n* = 8).

Where diet was measured, this was done using food frequency questionnaires (*n* = 3), and 24-h diet recalls (*n* = 3) followed by diet histories (*n* = 1) and other nutrition-related questionnaires. As for PA, this was self-reported via the Physical Activity Questionnaire for Children (PAQ-C) in nearly half of the studies (*n* = 8).

Five studies were conducted in urban areas and three took place in both urban and rural areas. The rest of the studies (*n* = 10) did not specify the location of the data collection. The majority of studies (*n* = 9) did not report maternal education; for the nine studies that did report on this maternal education, the majority of mothers had a secondary school level of education. Eight studies did not report on household income. The other ten studies reported that the majority of adolescents lived in households with a moderate level of household income. Gender was the most frequently adjusted covariate in only four studies [34,40,44,49]. Based on the quality assessment criteria, only one study was rated as good [34] while the others were rated as low quality (Table S1).

### 3.1. Dietary Determinants of Dietary Behaviours

Table 2 summarizes the associations between the determinants of diet and dietary behaviours. The factors related to dietary behaviours were investigated in nine studies. The outcomes were grouped into three categories: energy and nutrients, dietary patterns, and foods. Gender and ethnicity were the most investigated correlates of diet. “Dietary pattern” was the most commonly assessed dietary behaviour in association with diet.

#### 3.1.1. Energy and Nutrients

According to the included studies, the significant correlates of energy and nutrient intakes were gender, place of residence, and meal and snacking patterns.

• Energy Intake

Five studies investigated the association between energy intake and the following potential determinants of diet: gender, place of residence, meal and snacking patterns. Four of the five studies made no adjustment for confounders [37,38,39,48]. Only one of the five studies focused on the possibility of a link between energy intake and eating away from home but found no significant association [49]. The three studies that focused on the potential association between energy intake and gender reported that males consumed significantly more energy than female adolescents [37,38,48]. One study investigated the relationship between energy intake and place of residence and reported that energy intake was significantly higher among rural adolescents compared to their urban counterparts [48]. One study showed with the greater frequency of having snacks, the amount of energy consumed was greater [39].

• Macronutrient Intakes

Three studies investigated the association between macronutrient intakes of carbohydrates, protein, total fat and unsaturated and saturated fat, as well as some potential determinants of diet (i.e., meal and snacking pattern, gender and place of residence) [39,48,49]. Two of the three studies showed significant associations between macronutrient intakes of carbohydrates, proteins and fats with meal and snacking pattern [39,49] and gender and place of residence without adjustment for confounders [48]. However, fat intakes were not associated with place of residence [48].

It was also reported that male adolescents had significantly greater macronutrient intakes compared to females and that rural adolescents had higher carbohydrate and protein intakes than adolescents from urban areas [48]. In addition, there was a significant difference between the meal and snacking patterns and macronutrient intakes. Moreover, more frequent snack intakes led to more carbohydrate intake as compared to protein and fat intake [39]. Furthermore, one study did not show any significant association between eating away from home and carbohydrate and protein intakes while the energy-adjusted fat intake was significantly higher in more frequent eating away from home after adjusting for confounders such as gender, ethnicity, household income and body mass index (BMI) [49].

• Other Nutrients

One study examined unadjusted associations between fibre, sugar and dietary cholesterol with gender and adolescents’ place of residence [48]. The study found no association between the intake of sugar and fibre with gender and their place of residence. However, it did show that rural adolescents had a significantly higher intake of dietary cholesterol compared to their urban counterparts.

#### 3.1.2. Foods

In the reviewed studies, foods were grouped into two categories: SSBs and food groups.

• Sugar-Sweetened Beverage (SSB) Intake

One cross-sectional study focused on the association between SSB intake and ethnicity, gender and maternal education without adjusting for covariates. The study found no association with gender and maternal education. In this study based on unadjusted associations, only significant differences were observed for SSB intake with ethnicity, where SSB intake was highest among Malay adolescents followed by Indian and Chinese adolescents [36].

• Food Groups

One study examined the association between food groups and gender, in which the component scores of fish, fruit, vegetables, milk and milk products for the healthy eating index (HEI) were found to have significant mean and median differences between male and female adolescents without any adjustment for confounders [35]. However, there was no association between gender and the following food groups: cereals and grains, legumes, poultry, meat and eggs.

• Dietary Patterns

In the studies considered in this review, dietary patterns were grouped into five categories: diet quality, healthy, Western and local dietary pattern score, meal skipping behaviours, snacking and meal frequency. The significant correlates of dietary patterns included the availability of healthy foods, ethnicity, gender, age, self-efficacy for healthy eating, PA, eating out, fast food consumption, maternal education, snacking practices, nutritional supplement consumption, breakfast skipping, soft drink consumption, household income and eating companion.

• Diet Quality

Only one study looked for correlates of diet quality by using the HEI for Malaysia in order to evaluate the diet quality of Malaysian adolescents [35]. Without adjusting for covariates, the study found that five correlates (i.e., the availability of healthy foods, ethnicity, gender, age and self-efficacy for healthy eating) were significantly associated with diet quality while the frequencies of consuming breakfast, lunch and dinner were not associated with diet quality. The study also found that male adolescents had significantly lower mean composite scores on the HEI in comparison to females, indicating that males had a poorer diet quality [35]. In addition, Malay adolescents had a significantly lower composite HEI score compared to Indian adolescents [35]. The study also showed that there was a significant positive correlation between age and diet quality of the participants [35]. Moreover, the total score for self-efficacy for healthy eating was correlated weakly and positively with the diet quality of the adolescent participants [35]. Lastly, the study concluded that the low availability of healthy foods may have been contributed to the poor diet quality noted among the adolescents [35].

• Healthy, Western and Local Dietary Pattern Scores

One study investigated the associations between the dietary patterns and diet correlates of Malay and Chinese Malaysian adolescents [34]. The study found that Malay adolescents had significantly higher scores for the Western-based food pattern and the local-based food pattern, whereas the Chinese adolescents had higher scores for the healthy-based food pattern.

The multivariate analyses that were conducted by the study after adjusting for potential confounders revealed that, in the case of the Malay adolescents, age (Beta = 0.141, SE = 0.033; *p* < 0.001) and PA level (Beta = 0.142, SE = 0.036; *p* < 0.001) were positively associated with the healthy-based food pattern, whereas higher frequencies of eating out (away from home) (Beta = −0.088, SE = 0.036; *p* = 0.014) and fast food consumption (Beta = −0.166, SE = 0.081; *p* = 0.041) were negatively associated. Also, high frequencies of weekly breakfast skipping (Beta = 0.476, SE = 0.129; *p* < 0.001) and eating out (Beta = 0.109, SE = 0.036; *p* = 0.003) were positively associated with the Western-based pattern, whereas age (Beta = −0.136, SE = 0.033; *p* < 0.001) and household income (Beta = −0.078, SE = 0.027; *p* = 0.005) were negatively associated. Moreover, a higher frequency of daily snacking (*p* = 0.013) was positively associated with the local-based food pattern.

As for the Chinese adolescents, age (Beta = 0.165, SE = 0.029; *p* < 0.001), PA level (Beta = 0.10, SE = 0.024; *p* < 0.001) and maternal education level (Beta = 0.242, SE = 0.114; *p* = 0.035) showed positive associations with the healthy-based pattern, whereas high frequencies of eating out (Beta = −0.086, SE = 0.026; *p* = 0.001) and fast food intake (Beta = −0.223, SE = 0.068; *p* = 0.001) were negatively associated. Also, higher weekly frequencies of eating out (Beta = 0.072, SE = 0.026; *p* = 0.007), fast food intake (Beta = 0.156, SE = 0.068; *p* = 0.023), soft drink consumption (Beta = 0.080, SE = 0.035; *p* = 0.023), and daily snacking practice (Beta = 0.157, SE = 0.055; *p* = 0.004) were positively associated with the Western-based food pattern, whereas age (Beta = −0.084, SE = 0.029; *p* = 0.004) was negatively associated.

• Meal Skipping Behaviours

One study investigated the unadjusted association between meal skipping behaviour and dietary correlates [31] and found that ethnicity and eating companion were associated with meal skipping behaviours while living arrangement was not significantly associated. Those who usually skipped meals were commonly Malays; while more than half of the Chinese and Indian participants never skipped any meals, more than half of the Malays skipped at least one meal [31]. The results also indicated that those who had a meal companion (either ate with family or peers) had a lower probability of practising meal skipping behaviours; the majority of adolescents who had a meal companion never skipped any meals while one-fourth of those who ate alone skipped all three main meals in a day [31].

• Meal and Snacking Frequency

Two studies examined the effect of gender on the meal and snacking frequency and both found that gender was a significant correlate in unadjusted associations [35,37]. In one of the studies, adolescent girls had significantly higher snacking frequency compared to boys [37]. The other study reported that the frequency of breakfast intake was significantly higher among male adolescents compared to female [35].

### 3.2. Physical Activity Determinants of Physical Activity Behaviours

Table 3 summarizes the associations between the potential determinants of PA and PA behaviours. The factors related to PA determinants were investigated in 12 studies. The PA determinants were grouped into four categories: demographics, physical-environmental, social-environmental and behavioural. The PA score and PA level were the most common outcomes related to PA behaviour. Gender and ethnicity were the most commonly studied correlates.

#### 3.2.1. Demographic Determinants of Physical Activity

In the reviewed studies, eight potential demographic determinants of PA were considered: age, gender, ethnicity, maternal employment, paternal and maternal education, household income, household size and parents’ marital status.

• Age

Two studies investigated the association between age and PA [40,44]. One of the studies showed that there was no significant association [44]. In the other study, after adjusting for confounders (i.e., age, gender, breakfast intake, BMI and school session), the results indicated that each additional year of age raised the odds of being physically inactive (OR = 1.2 (95% CI: 1.16–1.23); *p* < 0.001) and thus younger adolescents were more physically active [40].

• Gender

Ten studies [27,37,38,40,41,42,43,44,46,47] focused on the association between gender and PA and level of PA. Significant gender differences in PA behaviour were reported in nine studies. Six studies found a consistent positive association between gender and PA and reported that boys were significantly more physically active than girls [37,40,41,42,44,47]. However, among these, only two studies made adjustments for confounders [40,44]. In one study, there was no significant association between gender and PA duration while girls reported spending longer each day on PA before and during school [46]. In contrast, two studies reported that adolescent boys had significantly higher daily PA levels and moderate to vigorous physical activity levels compared to girls [38,43]. In the cohort study, PA among girls residing in rural areas dropped significantly in a cohort study from baseline in 2012 to the first follow-up in 2014 [27].

• Ethnicity

Five studies [34,41,42,44,47] examined the association between ethnicity and PA. The significant associations between ethnicity and PA behaviour were consistent in four studies [34,41,44,47], but the associations were adjusted for covariates in only one of these studies [44]. In one of the studies, race was one of the factors that differed significantly between the active and inactive group (Malays vs others), where Malays were more active than other ethnicities [41]. In another study, the PA scores of the Indian adolescents were slightly lower followed by the Malays. In contrast, it has been reported that Chinese adolescents were the least active [47]. In addition, it was reported in another study that, compared to Malays, Chinese spend less time in PA [44].

• Maternal Employment

One study reported a significant association between PA behaviour and maternal employment [41] Based on the result of a logistic regression analysis, one study reported that adolescents with an unemployed mother were more likely to be physically inactive compared to those with an employed mother (OR = 2.167 (95% CI: 1.263–3.717); *p* = 0.005) [41].

• Paternal and Maternal Education

Two studies focused on the associations between PA and paternal and maternal education [42,44]. In one study, there was a positive but weak association between paternal education and PA score [42].

The other two studies did not find any association with paternal education. Only one study out of three found that adolescents who had a primary- or secondary-educated mother were less likely to participate in PA compared to adolescents who had a tertiary-educated mother [44].

• Household Income

One study examined the association between PA and household income, and did not find any association [42].

• Household Size

Two studies focused on household size and PA [42,44]. One study did not show any significant associations [42]. The results of the other study showed that household size could increase the likelihood of being physically active. Specifically, at the conditional level, an additional household member increased the time spent by the adolescent on PA by 0.083 days. At the unconditional level, an increase in household size raised the time spent on PA by 0.062 days. The results also showed that there was an approximately 0.9% increase (based on the estimated coefficients) in the probability of participating in PA when the household size increased by one member [44].

• Parents’ Marital Status

One study looked at the association between parents’ marital status and PA, and did not find any association [44].

#### 3.2.2. Physical-Environmental Determinants of Physical Activity

The physical-environmental determinants of PA that were considered by the studies included in this systematic review were school session and place of residence, as well as a number of environmental barriers to the use of PA facilities, namely, hot weather, availability of equipment, distance between facility and home, usage level of facilities and facility support.

• School Session

One study, after controlling for other factors, indicated that adolescents attending afternoon school sessions had higher odds of being physically inactive than those attending morning sessions (AOR = 1.3; 95% CI: 1.13–1.44) [40].

• Place of Residence

Two studies related to one cohort study examined the association between place of residence and physical activity without adjustment for covariates [27,47]. One did not find any significant association at the cross-sectional level [47] but observed a downward trend in the PA level among all adolescents with a significant reduction among all rural students from 2012 (baseline) to 2014 (first follow-up) [27].

• Environmental Barriers to Use of PA Facilities

Three studies examined the association between environmental barriers and the use of PA facilities without any adjustment for covariates [41,45,46]. One study did not show any associations [46]; however, one study showed a significant association with environmental barriers [41] and one identified significant associations with the environmental characteristics of the facilities themselves [45]. In one study, it was reported that there were significant differences between the active and inactive group with regards to the effect of personal environmental factors such as hot weather, availability of equipment and distance between facility and home, which were categorized as barriers to PA [41]. In the other study, it was found that facility support and usage level of facilities had a significant but poor relationship with adolescents’ involvement in physical activities [45].

#### 3.2.3. Social-Environmental Determinants of Physical Activity

Three studies examined the social-environmental determinants of PA, namely, family without exercise, family and peer influence and physical education [41,42,44].

• Family Without Exercise

One study indicated that PA was significantly lower among adolescents who lived in a family without exercise [41].

• Family and Peer Influence

One study showed that there was a positive and significant relationship between peer influence and PA score without adjustment for covariates [42]. It also showed that family influence had a positive but weak impact on PA score.

• Physical Education

One study reported that after adjusting for covariates (age, gender, and ethnicity) an additional day spent in attending a physical education class increased the time spent on PA by 0.200 (unconditional) and 0.151 (conditional) days. The probability of participating in PA also increased by 2.2% when the time spent attending physical education class increased by one day [44].

#### 3.2.4. Behavioural Determinants of Physical Activity

Two studies evaluated the behavioural determinants of PA [40,41]: one considered breakfast intake, while the other considered the effects of six personal factors: preferring to watch TV, being embarrassed, being lazy, finding exercise too troublesome, only exercising when having ample time, and seeing stretching as important before exercise.

• Breakfast Intake

The study on breakfast intake and PA, which made adjustments for covariates, found that the odds of adolescents who did not consume breakfast being inactive increased 1.9 times, which was considered significant (AOR = 1.9; 95% CI: 1.74–2.13) and among those who had irregular breakfast intake the odds increased by 1.4 times (AOR = 1.9; 95% CI: 1.33–1.55) compared to those who consumed breakfast daily [40].

• Personal Barriers

The study that examined several personal barriers to PA did not adjust for covariates. The results of the study showed that there were significant differences between the active and inactive group with regards to the following personal factors: stretching is important before exercise, exercise when having ample time, prefer to watch TV, embarrassed, being lazy and too troublesome [41]. In addition, the time constraint was associated with inactivity, where those who reported that time constraints prevented them from doing PA were 2.5 times more at risk of becoming inactive. Those who stated that they preferred to do exercise when the time was available were 2.5 times more likely to be inactive and those who stated that stretching was necessary before exercise were 3.7 times more likely to be inactive [41].

## 4. Discussion

This systematic review identified 18 studies on the potential factors influencing the dietary and PA behaviours of Malaysian adolescents. This review clearly demonstrates that for many variables, the evidence is insufficient and this is mainly due to the limited number of studies. Also, the associations that were found were often small or inconsistent, with few studies controlling for confounding factors. Many of the factors identified by this systematic review have been reported in other systematic reviews [2,50,51]. This review found that most of the diet-related studies focused on dietary patterns and nutrient analysis, while only a few of them reported results concerning specific food groups. The most common PA-related outcomes identified by this review were PA score and PA intensity. It was not possible to establish strong relationships between some of the significant associated diet/PA correlates and diet/PA behaviours because these were evaluated in only one study.

The finding of this systematic review with respect to gender differences in dietary intake suggest that male adolescents consumed more energy [37,38,48] and macronutrients [48] compared to females while the intakes of carbohydrate and protein were higher among rural adolescents than their urban counterparts. In addition, male adolescents had a lower diet quality [35], snacking frequency [35] and higher meal frequency than females [37]. This finding is in agreement with a previous systematic review, which reported that most girls had a desire to be thin as they perceived that a thin figure is the ideal female body image. It also found that girls had higher nutrition knowledge scores than boys, particularly because girls were more likely to read nutritional food labels [52].

The previous systematic review also revealed that many other factors such as PA level, socioeconomic status (SES), diet, individual and social factors contribute to either a higher or a lower energy intake among adolescents [52]. In regards to such factors, this review only found weak evidence for the relation between ethnicity and eating and meal skipping behaviours. Specifically, the studies included in this review showed that the adolescents who usually skipped meals were Malay or those who usually ate alone [31]. A previous systematic review indicated that there was a significant positive association between peer influence and eating habits, meaning that the higher the peer pressure, the unhealthier the students’ dietary intake [52]. The previous review also suggested that the presence of peers and friends increases the energy intake of children and adolescents [52].

In this systematic review, only weak evidence was found for the effect of ethnic differences on dietary patterns, where Malays had higher SSB consumption [36] and lower diet quality than Indian and Chinese adolescents [35]. In addition, one of the reviewed studies showed that Malay adolescents were more likely to have Western-based and local-based dietary patterns, whereas Chinese adolescents were more likely to follow a healthy-based food pattern [34]. An earlier systematic review indicated that the dietary habits of ethnic populations can be affected by many factors such as the availability of food, income level, food beliefs, religion, cultural patterns and customs [53]. Thus, the ethnic differences in eating patterns may possibly be a reflection of socio-cultural differences related to food preferences [54].

This systematic review also found that age (being older) and higher maternal educational status were associated with a healthy-based food pattern among Malaysian adolescents [34]. On the other hand, from the reviewed studies it was found that Malay adolescents in lower-income households had a higher intake of Western-based food [34]. Thus this review presents consistent evidence that SES factors (e.g., parental education level, household income) were associated with dietary behaviours, which is in line with the findings of other systematic reviews [51,55]. However, a previous systematic review determined that interpersonal factors also had a major role to play in dietary behaviours, whereas school, neighbourhood or societal factors were not consistently associated with dietary behaviours [56].

In addition, it was reported in another systematic review that adolescents with low SES may have poorer dietary patterns compared to high-SES adolescents because low-SES families may not be able to buy nutritious foods [57]. Moreover, the review argued that it is possible that low-SES families have less information of the nutritional content and the daily recommendations of food groups [58]. Furthermore, low-SES families may have more fast food consumption and have a lower intake of healthy options [59]. A systematic review of studies related to high, medium and low development countries also suggested that, as countries develop economically, there is a higher adherence to an unhealthy diet in adolescents of parents with lower education level [60].

The results of this systematic review suggest that the significant correlates of PA for adolescents, with limited evidence, are gender (male), ethnicity (Malay), paternal and maternal education (higher), household size (bigger), family and peer influence and physical education. The reviewed studies showed that higher PA intensity was related to gender (male). In addition, physical inactivity was found to be significantly associated with age (older), breakfast (no intake), school session (afternoon), personal barriers (e.g., family without exercise, unemployed mother), environmental barriers (e.g., hot weather, unavailability of equipment and distance between facility and home) and place of residence (rural).

One consistent finding of this systematic review, which is similar to that reported in other systematic reviews, was that boys are more active than girls and that PA decreases with increasing age. On the other hand, the results reported in this review regarding the influence of ethnicity show that it correlates with adolescents’ PA [61], whereas in the other systematic review the evidence was inconclusive [62]. In this review, the evidence related to the effect of age on PA was weak; however, there was a relationship.

The above findings of this review are consistent with those presented in a previous review, which identified that cluster patterns were different among adolescents according to age and gender, where a higher proportion of girls and older adolescents were in the clusters defined by low levels of PA [2]. A reduction in PA related to age during adolescence has also been reported in a recent systematic review of European children and adolescents [63]. This decrease in PA may be due to the fact that older adolescents have to focus more on academic activities and achievements. In the Malaysian context, adolescents (13–18 years) in middle/high school are under pressure to pass high school/college entrance examinations. In addition, it has been indicated in other studies that female adolescents were more concerned about others seeing their bodies while performing PA. They were also less interested in vigorous PA, which was more favoured by males [64]. In addition, girls were more responsible for doing housework, thereby limiting the time they had for PA [65].

Furthermore, this review found weak evidence that school session (afternoon session) and breakfast intake (no breakfast and irregular breakfast) were significant factors related to physical inactivity among Malaysian adolescents [40]. It was explained in one of the reviewed studies that skipping breakfast can lead to mental and physical fatigue as well as energy and nutrient deficiencies that affect the ability to perform physical activities [66].

Finally, it was indicated in a previous systematic review that PA in adolescents was unrelated to the copying of friends and siblings, support of parents for PA, and family size and that instead it was more consistently related to school and neighbourhood characteristics than to interpersonal and societal environments [56]. However, in another systematic review, it was indicated that most studies on the environmental determinants of PA rely on self-reports of environmental factors and thus represent the perceived, rather than the actual features of the physical environment [51].

### 4.1. Limitations

However, some issues and limitations should be considered when interpreting the findings. First, all of the included studies except one were cross-sectional and cannot be considered good-quality, thus the conclusions about the direction and possible associations may not be precise. Second, the majority of studies relied on self-reported data, and it was difficult to judge the validity and reliability of the PA and diet measurement tools that used to collect these data because this information was not reported in several of the studies and some used invalid or poorly validated tools. Also, the variation in the age of the adolescents involved in the reviewed studies may also have interfered with the appropriateness of the methods used for measuring exposures and outcomes and may also have affected the magnitude of the errors in the information and measurements.

In addition, the literature reviewed presented very heterogeneous and inconsistent results with regard to the correlates of diet and of PA in adolescents. Furthermore, this inconsistency remained even when the results of previous reviews were compared with those of this review. Possible reasons for this are the lack of consistency between studies in regards to the methods used for measuring dietary intake and PA and the presence of methodological errors as well as the use of inaccurate and imprecise techniques. For instance, in the reviewed studies only questionnaires were used to assess PA; no accelerometers or other related electronic devices were used. Furthermore, it is notable that one previous systematic review concluded that no questionnaires with acceptable reliability and validity were available for the assessment of PA among adolescents [67].

Also, in this systematic review, in order to compile and analyse the results of the included studies, conceptually similar determinants were placed into the same category, even though some of the potential determinants in the same category were often different or measured in different ways. Insufficient control for confounders also appeared to be a problem in the majority of studies. For instance, in some studies, the analyses were not adjusted for relevant socio-demographic confounders such as gender, age and/or SES.

Additionally, many studies used samples that were non-representative or only representative of a limited geographical area. The majority of studies focused on specific states such as Kuala Lumpur and Selangor and Pahang, whereas only a few of the included studies were conducted in the northeast regions of Malaysia. This indicates that the development of scientific research on the diet/PA behaviours of Malaysian adolescents has yet to cover all regions of the country.

Lastly, the reviewed studies were heterogeneous in terms of their measurement, samples and analyses, and therefore it was not probable to evaluate the overall strength of the associations. Thus, an improvement in the quality of further studies could lead to more consistent studies and greater sureness in the identified correlates and determinants of diet and PA.

### 4.2. Recommendations for Further Research

Regardless of the heterogeneity in the measures, specific behaviours and study areas, this review was able to highlight the factors that were or were not related to PA and dietary behaviours in Malaysian adolescents. It is therefore hoped that the results of this review will help in identifying potentially effective mediators that can be used in interventions to promote a healthy lifestyle among this population group. The results suggest that interventions could incorporate structured physical activities before and after school or during breaks; improved accessibility of PA facilities nearby the school environment; increased physical education and PA sessions; improved availability of healthy food options especially in school canteens and government-subsidized healthy foods in schools. Currently, the Malaysian government only subsidizes foods in primary schools. However, extending this subsidy to high schools may prepare more opportunities for low-SES adolescents to eat healthy foods. Despite there are some studies that is available and mostly well conducted from the higher income countries, some of the policy that was developed for healthy lifestyle may not be suitable to be adapted in Malaysian local context. It has been stated in a systematic review the main structural factors that affect adolescent health include economic and political systems, the education system, wealth and its distribution within a country, poverty, migration and cultural factors such as gender and ethnic equality, as well as factors such as climate change and war or conflict [68]. National wealth and income dissemination affect adolescent-health outcomes across countries. There is considerable evidence that income inequality within countries has the impact on many aspects of adolescent health, principally among middle-income and high-income countries [24].

Another systematic review also revealed that in low-income countries or in countries with low human development index (HDI), the relation between SES and obesity seems to be positive for both men and women: the wealthier and/or those with higher educational level tend to be more likely to be obese. However, in middle-income countries or in countries with medium HDI, the relation becomes mostly mixed for men and negative for women [69].

School-based interventions have the potential to improve adolescents’ dietary and PA behaviours because adolescent students spend on average at least six hours in school per day during term-time. Potentially the healthy lifestyle subjects should be incorporated in a more interactive curriculum/ manner. The availability of well maintain playing area/ park in addition to the availability of healthy food is crucial to ensure PA program can be conducted. It is also recommended that gender-specific strategies are identified for use in further interventions to improve the eating patterns and PA among Malaysian adolescents. Lifestyle and health-related interventions that focus on PA and healthy eating practices require attention from all the stakeholders. High-quality longitudinal studies using electronic devices to assess daily PA is also needed to improve understanding of the dietary and PA behaviours of Malaysian adolescents.

## 5. Conclusions

This systematic review is the first to summarize the determinants associated with diet and PA behaviours among adolescents in Malaysia. However, the significance of these associations was often small or inconsistent. This review highlights there is a lack of longitudinal observational research to support the causal role of specific factors in improving diet and PA behaviour in Malaysian adolescents. However, this review summarizes the best available evidence for local policymakers and public health practitioners so that they can consider incorporating these findings into the development of intervention protocols for improving diet, PA and health in Malaysian adolescents.

## Figures and Tables

**Table 1:** Characteristics of the included studies.

Author, Year [Ref]	Setting/Urbanity	Sample	Age (y) Mean ± SD	Ethnicity	Maternal Education	Income (RM)	Diet Measure	Diet Outcome	PA Measure	PA Outcome	Covariates
Chin & Mohd Nasir 2009 [31]	Kuantan in Pahang/NR	407 (♀)	15.2 ± 1.9	Malay, Chinese, Indian	secondary: 57.0%	Mean ± SD RM 3266 ± 2566	OQ (EBQ)	Meal skipping behaviours	NA	NA	NR
Abdullah et al. 2016 [34]	Kelantan/NR	454 (♂ ♀)	15.3 ± 1.9	Malay, Chinese	Malay; secondary: 67.8% Chinese; secondary: 72.5%	Malay: 70% Chinese: 68% low income (<RM 2300)	FFQ	Healthy, Western & Local dietary pattern score	PAQ-C	PA	Age, gender, ethnicity, SES, breakfast skipping, snacking, eating out, fast food intake, soft drink intake, dietary supplement, PA levels, screen viewing
Rezali et al. 2015 [35]	Kuala Lumpur/Urban	373 (♂ ♀)	14.3 ± 1.2	Malay, Chinese, Indian, other	NR	NR	2 × 24R & OQ (EBQ)	Diet quality, food groups, meal frequency	NA	NA	NR
Loh et al. 2017 [36]	Kuala Lumpur/Urban	873 (♂ ♀)	13 *	Malay, Chinese, Indian, other	Secondary: 61.4%	NR	OQ (CNQ)	Sugar sweetened beverages intake	NA	NA	NR
Nurul-Fadhilah et al. 2013 [37]	Kota Bharu in Kelantan/NR	236 (♂ ♀)	15.3 ± 1.9	Malay	NR	(Mean ± SD) RM 2191 ± 2553	FFQ	Energy intake, frequency of eating out, snacking frequency	PAQ-C	PA	NR
Teo et al. 2014 [38]	Kota Bharu in Kelantan/NR	454 (♂ ♀)	15.3 ± 1.9	Malay, Chinese	NR	NR	FFQ	Energy intake	PAC-C	PA & MVPA	NR
Boon et al. 2012 [39]	Kuala Lumpur/Urban	156 (♂ ♀)	14.1 ± 0.8	Malay, Chinese, Indian	NR	Moderate (RM 2000–5999): 57.1%	1 × 24R	Energy & macronutrients intake	NA	NA	NR
Abdul Majid et al. 2016 [48] *	Kuala Lumpur, Selangor, Perak/Urban & rural	794 (♂ ♀)	12.86 ± 0.3	Malay, Chinese, Indian, other	Secondary: 66%	Low SES: 49%	7DH	Energy & macronutrients intake	NA	NA	NR
Cynthia et al. 2013 [49]	Puchong in Selangor/Urban	408 (♂ ♀)	13.74 ± 0.56	Malay, Chinese, other	Upper secondary: 38.9%	41.9% < RM 3999	2 × 24R	Energy & macronutrients intake	NA	NA	Gender, ethnicity, BMI
Baharudin et al. 2014 [40]	National/NR	40011 (♂ ♀)	13.48 ± 2.24	NR	NR	NR	NA	NA	PAQ-C	Physical inactivity	Age, gender, breakfast intake, BMI, School session
Aniza et al. 2009 [41]	Petaling in Selangor/Urban	519 (♂ ♀)	14 and 16	Malay, Chinese, Indian	NR	Father—41.6% Mother—80.9% Low income (<RM 1500)	NA	NA	IPAQ	Physical inactivity, PA	NR
Dan et al. 2011 [42]	Kuantan in Pahang/NR	400 (♂ ♀)	13.23 ± 0.31	Malay, Chinese, Indian	Total years (Mean ± SD): 12.29 ± 3.39	>RM 3000: 38.5%	NA	NA	PAQ-C	PA	NR
Farah Wahida et al. 2011 [43]	Kuantan in Pahang/NR	360 (♂ ♀)	13.2 ± 0.3	Malay, Chinese, Indian, other	Secondary: 50.6%	NR	NA	NA	PAQ-C	PA level, MVPA	NR
Cheah et al. 2016 [44]	NR/NR	2991 (♂ ♀)	15.88 ± 0.71	Malay, Chinese, Indian, other	Secondary: 64.39%	NR	NA	NA	OQ	PA	Age, gender, ethnicity
Abd-Latif et al. 2012 [45]	Seremban, Muar, Kota, Star, Kuantan/NR	913 (♂ ♀)	13–17	NR	NR	Medium SES: 44%	NA	NA	OQ	PA involvement	NR
Cheah et al. 2012 [46]	Kuching in Sarawak/NR	316 (♂ ♀)	14–16	Malay, Chinse	Secondary: 63.9%	Mean ± SD RM 3652.9 ± 3740.6	NA	NA	OQ	PA	NR
Su et al. 2014 [47] *	Kuala Lumpur, Selangor, Perak/Urban & rural	1327 (♂ ♀)	12.9 ± 0.3	Malay, Chinese, Indian, other	NR	NR	NA	NA	PAQ-C	PA	NR
Abdul Majid et al. 2016 [27] *	Kuala Lumpur, Selangor, Perak/Urban & rural	820 (♂ ♀)	15	Malay, Chinese, Indian, other	NR	NR	NA	NA	PAQ-C	PA	NR

Note: ♂, Male; ♀, Female; SES, Socio-Economic Status; NR, Not Reported; NA, Not Available; RM, Malaysian Ringgit (currency); MVPA, Moderate to Vigorous Physical Activity; BMI, Body Mass Index; FFQ, Food Frequency Questionnaire; PA, Physical Activity; X, Number; X × 24R, 24-h Recall completed over X days; XDH, X days Diet History; OQ, Other Questionnaire; EBQ, Eating Behaviours Questionnaire; CNQ, Child Nutrition Questionnaire; PAQ-C, Physical Activity Questionnaire for Children; *, MyHeARTs study.

**Table 2:** Summary of associations between the determinants of diet and dietary behaviours.

Author, Year [Ref]	Outcome	Correlate	Association	*p*-Value
Energy & Nutrients				
Teo et al. 2014 [38]	Energy intake (kcal/day)	Gender	Male vs. Female (Median (95%, CI)	
			Malay ♂ 2408 (2255–2437) vs. ♀ 2178 (2058–2246)	*p* < 0.01
			Chinese ♂ 1860 (1792–1970) vs. ♀ 1649 (1642–1828)	*p* < 0.05
Abdul Majid et al. 2016 [48]			Male vs. Female (Mean (95% CI) ♂ 1774.0 (1730.8–1817.3) vs. ♀ 1595.2 (1567.4–1623.1)	*p* < 0.001
Nurul-Fadhilah et al. 2013 [37]			Male vs. Female (Mean ± SD) ♂ 2346 ± 468 vs. ♀ 2152 ± 547	*p* < 0.01
Abdul Majid et al. 2016 [48]		Place of residence	Urban vs. Rural (Mean (95% CI) 1612.3 (1581.6–1643.1) vs. 1706.1 (1668.7–1743.4)	*p* < 0.001
Boon et al. 2012 [39]		Meal patterns	(Mean ± SD) 3M + 3S: 1952 ± 411 vs. 3M + 2S: 1883 ± 456 vs. 3M + 1S: 1687 ± 426 vs. 3M: 1405 ± 426 vs. ≤2M + 2,3S: 1414 ± 335 vs. ≤2M + 0,1S: 1340 ± 252	*p* < 0.05
		Snacking patterns	(Mean ± SD) 3M + 3S: 793 ± 246 vs. 3M + 2S: 514 ± 207 vs. 3M + 1S: 259 ± 176 vs. 3M vs. ≤2M + 2: 259 ± 176 vs. 3S: 0 vs. ≤2M + 0: 459 ± 209 vs. 1S: 247 ± 244	*p* < 0.05
Cynthia et al. 2013 [49]		Eating out	0–2 times vs. 3–6 times vs. ≥7 times (Mean ± SE) 1984 ± 65 vs.1915 ± 97 vs. 2077 ± 100	*p* = NS
Abdul Majid et al. 2016 [48]	Carbohydrate intake (g/day)	Gender	Male vs. Female (Mean (95% CI) ♂ 245.2 (238.6–251.8) vs. ♀ 220.0 (215.7–224.2)	*p* < 0.001
		Place of residence	Urban vs. Rural (Mean (95% CI) 221.6 (216.8–226.3) vs. 236.4 (230.8–242.0)	*p* < 0.001
Cynthia et al. 2013 [49]		Eating out	0–2 times vs. 3–6 times vs. ≥7 times (Mean ± SE) 131.10 ± 1.69 vs. 132.02 ± 2.03 vs. 126.9 ± 2.58	*p* = NS
Boon et al. 2012 [39]		Meal pattern	(Mean ± SD) 3M + 3S: 258.9 ± 49.5 vs. 3M + 2S: 253.7 ± 72.6 vs. 3M + 1S: 225.2 ± 64.1 vs. 3M: 187.0 ± 40.0 vs. ≤2M + 2,3S: 200.2 ± 50.8 vs. ≤2M + 0,1S: 168.1 ± 43.7	*p* < 0.05
		Snacking practices	(Mean ± SD) 3M + 3S: 111.5 ± 34.4 vs. 3M + 2S: 74.0 ± 29.8 vs. 3M + 1S: 37.0 ± 24.8 vs. 3M: 0 vs. ≤2M + 2,3S: 71.8 ± 28.5 vs. ≤2M + 0,1S: 38.4 ± 37.9	*p* < 0.05
Abdul Majid et al. 2016 [48]	Protein intake (g/day)	Gender	Male vs. Female (Mean (95% CI) ♂ 65.7 (63.6–67.7) vs. ♀ 58.7 (57.4–59.7)	*p* < 0.001
		Place of residence	Urban vs. Rural (Mean (95% CI) 59.3 (57.9–60.6) vs. 63.1 (61.1–64.8)	*p* = 0.001
Cynthia et al. 2013 [49]	Protein intake (g/day)	Eating out	0–2 times vs. 3–6 times vs. ≥7 times (Mean ± SE) 42.37 ± 0.74 vs. 40.08 ± 0.89 vs. 43.73 ± 1.13	*p* = NS
Boon et al. 2012 [39]		Meal pattern	(Mean ± SD) 3M + 3S: 79.7 ± 21.4 vs. 3M + 2S: 73.7 ± 17.9 vs. 3M + 1S: 72.1 ± 26.2 vs. 3M: 58.7 ± 15.8 vs. ≤2M + 2,3S: 49.7 ± 12.4 vs. ≤2M + 0,1S: 54.5 ± 14.4	*p* < 0.05
		Snacking practices	(Mean ±SD) 3M + 3S: 25.4 ± 10.3 vs. 3M + 2S: 16.4 ± 8.7 vs. 3M + 1S: 8.1 ± 9.60 vs. 3M: 0 vs. ≤2M + 2,3S: 11.3 ± 8.1; ≤2M + 0,1S: 8.1 ± 11.5	*p* < 0.05
Cynthia et al. 2013 [49]	Fat (g/day)	Eating out	0–2 times vs. 3–6 times vs. ≥7 times (Mean ± SE) 34.05 ± 0.64 vs. 34.54 ± 0.77 vs. 34.86 ± 0.98	*p* = 0.043
Boon et al. 2012 [39]		Meal pattern	(Mean ± SD) 3M + 3S: 66.7 ± 21.4 vs. 3M + 2S: 64.3 ± 21.2 vs. 3M + 1S: 55.3 ± 18.2 vs. 3M: 47.1 ± 17.0 vs. ≤2M + 2,3S: 46.4 ± 18.0 vs. ≤2M + 0,1S: 50.0 ± 15.9	*p* < 0.05
		Snacking practices	(Mean ± SD) 3M + 3S = 27.7 ± 14.1 vs. 3M + 2S = 17.2 ± 10.3 3M + 1S = 8.8 ± 7.6 vs. 3M = 0 vs. ≤2M + 2 vs. 3S = 14.4 ± 10.6 vs. ≤2M + 0,1S = 6.9 ± 8.2	*p* < 0.05
Abdul Majid et al. 2016 [48]		Gender	Male vs. Female (Mean (95% CI) ♂ 59.7 (57.6–61.7) vs. ♀ 53.2 (52.0–54.3)	*p* < 0.001
		Place of residence	Urban vs. Rural (Mean (95% CI) 54.6 (53.2–56.0) vs. 56.4 (54.8–57.9)	*p* = NS
Abdul Majid et al. 2016 [48]	Cholesterol (mg/d)	Gender	Male vs. Female (Mean (95% CI) ♂ 248.8 (236.5–261.0) vs. ♀ 209.1 (201.6–216.7)	*p* < 0.001
		Place of residence	Urban vs. Rural (Mean (95% CI) 202.6 (194.2–211.1) vs. 244.1 (234.1–254.0)	*p* < 0.001
	Mono-unsaturated fatty acid (g/d)	Gender	Male vs. Female (Mean (95% CI) ♂ 9.0 (8.5–9.5) vs. ♀ 7.9 (7.6–8.1)	*p* < 0.001
		Place of residence	Urban vs. Rural (Mean (95% CI) 8.3 (7.9–8.6) vs. 8.3 (7.9–8.6)	*p* = NS
	Poly-unsaturated fatty acid (g/d)	Gender	Male vs. Female (Mean (95% CI) ♂ 6.4 (6.1–6.7) vs. ♀ 5.8 (5.6–6.0)	*p* = 0.005
		Place of residence	Urban vs. Rural (Mean (95% CI) 5.9 (5.7–6.2) vs. 6.2 (5.9–6.4)	*p* = NS
	Saturated fatty acid (g/d)	Gender	Male vs. Female (Mean (95% CI) ♂ 12.0 (11.2–12.7) vs. ♀ 10.3 (9.9–10.7)	*p* < 0.001
		Place of residence	Urban vs. Rural (Mean (95% CI) 10.8 (10.4–11.3) vs. 10.9 (10.4–11.5)	*p* = NS
	Sugar (g/d)	Gender	Male vs. Female (Mean (95% CI) ♂ 34.7 (32.5–36.8) vs. ♀ 34.1 (32.7–35.5)	*p* = NS
		Place of residence	Urban vs. Rural (Mean (95% CI) 34.1 (32.3–35.8) vs. 34.5 (33.0–36.1)	*p* = NS
Abdul Majid et al. 2016 [48]	Crude fiber (g/d)	Gender	Male vs. Female (Mean (95% CI) ♂ 2.9 (2.7–3.1) vs. ♀ 3.0 (2.8–3.1)	*p* = NS
		Place of residence	Urban vs. Rural (Mean (95% CI) 3.0 (2.8–3.1) vs. 2.9 (2.7–3.1)	*p* = NS
Foods				
Rezali et al. 2015 [35]	Cereals and grains (HEI score)	Gender	Male vs. Female (Mean ± SD) ♂ 5.5 ± 1.9 vs. ♀ 5.4 ± 2.1	*p* = NS
	Fish (HEI score)		Male vs. Female (Mean ± SD) ♂ 1.6 ± 2.0 vs. ♀ 3.4 ± 3.6	*p* < 0.05
	Fruit (HEI score)		Male vs. Female (Median) ♂ 0 vs. ♀ 0	*p* < 0.05
	Legumes (HEI score)		Male vs. Female (Mean ± SD) ♂ 1.8 ± 2.9 vs. ♀ 1.6 ± 2.6	*p* = NS
	Vegetables (HEI score)		Male vs. Female (Mean ± SD) ♂ 3.7 ± 2.5 vs. ♀ 3.1 ± 2.4	*p* < 0.05
	Poultry, meat & egg (HEI score)		Male vs. Female (Mean ± SD) ♂ 8.0 ± 2.9 vs. ♀ 8.6 ± 2.6	*p* > 0.05
	Milk and milk products (HEI score)		Male vs. Female (Median) ♂ 0 vs. ♀ 1	*p* < 0.05
Loh et al. 2017 [36]	Sugar sweetened beverages (SSB) (mL/day)	Ethnicity	Malay vs. Chinese vs. Indian vs. others (mean ± SE) 0.76 ± 0.04 vs. 0.44 ± 0.05 vs. 0.55 ± 0.10 vs. 0.63 ± 0.21	*p* = 0.03
		Gender	Male vs. Female (mean ± SE) ♂ 0.68 ± 0.08 vs. ♀ 0.67± 0.03	*p* = NS
		Maternal education	Primary vs. Secondary vs. Tertiary (mean ± SE) Primary: 0.62 ± 0.07 vs. Secondary: 0.74 ± 0.05 vs. Tertiary: 0.61 ± 0.06	*p* = NS
Dietary Patterns				
Rezali et al. 2015 [35]	Diet quality (HEI score)	Availability of healthy foods	Beta = 0.351	*p* < 0.05
		Ethnicity	Malay; Beta = −2.416	*p* < 0.05
		Gender	Male vs. Female (Mean ± SD) ♂ 34.2 ± 8.2 vs. ♀ 39.9 ± 9.0; Beta ♂ = −5.883	*p* < 0.05
		Age	r = 0.123	*p* < 0.05
		Self-efficacy for healthy eating	Beta = 0.242	*p* < 0.05
		Frequency of breakfast	r = 0.038	*p* = NS
Abdullah et al. 2016 [34]	Healthy dietary pattern score	Age	Malay vs. Chinese	
			Beta = 0.141, SE = 0.033	*p* < 0.001
			Beta = 0.165, SE = 0.029	*p* < 0.001
		PA	Malay vs. Chinese	
			Beta = 0.142, SE = 0.036	*p* < 0.001
			Beta = 0.10, SE = 0.024	*p* < 0.001
		Eating out	Malay vs. Chinese	
			Beta = −0.088, SE = 0.036	*p* = 0.014
			Beta = −0.086, SE = 0.026	*p* = 0.001
		Ethnicity	Malay vs. Chinese (Mean ± SD) −0.101 ± 0.957 vs. 0.094 ± 1.03	*p* = 0.039
		Fast food consumption	Malay vs. Chinese	
			Beta = −0.166, SE = 0.081	*p* = 0.041
			Beta = −0.223, SE = 0.068	*p* = 0.001
		Maternal education	Chinese; Beta = 0.242, SE = 0.114	*p* = 0.035
	Local dietary pattern score	Eating out	Chinese; Beta = 0.067, SE = 0.022	*p* = 0.003
		Fast food consumption	Chinese; Beta = 0.133, SE = 0.057	*p* = 0.021
		Snacking practices	Malay vs. Chinese	
			Beta = 0.158, SE = 0.063	*p* = 0.013
			Beta = 0.254, SE = 0.096	*p* = 0.009
		Ethnicity	Malay vs. Chinese (Mean ± SD) 0.399 ± 1.05 vs. −0.427 ± 0.73	*p* < 0.001
		Nutritional supplements consumption	Chinese; Beta = −0.216, SE = 0.097	*p* = 0.027
	Western dietary pattern score	Breakfast skipping	Malay; Beta = 0.476, SE = 0.129	*p* < 0.001
		Eating out	Malay vs. Chinese	
			Beta = 0.109, SE = 0.036	*p* = 0.003
			Beta = 0.072, SE = 0.026	*p* = 0.007
		Fast food consumption	Chinese; Beta = 0.156, SE = 0.068	*p* = 0.023
		Snacking practices	Chinese; Beta = 0.157, SE = 0.055	*p* = 0.004
Abdullah et al. 2016 [34]	Western dietary pattern score	Soft drink consumption	Chinese; Beta = 0.080, SE = 0.035	*p* = 0.023
		Household income	Malay; Beta = −0.078, SE = 0.027	*p* = 0.005
		Age	Malay vs. Chinese	
			Beta = −0.136, SE = 0.033	*p* < 0.001
			Beta = −0.084, SE = 0.029	*p* = 0.004
		Ethnicity	Malay vs. Chinese (Mean ± SD) 0.224 ± 1.04 vs. −0.239 ± 0.89	*p* < 0.001
Nurul-Fadhilah et al. 2013 [37]	Frequency of eating out (times/week)	Gender	Male vs. Female (%) Daily: 8 vs. 7 4–6 times/week: 22 vs.32 1–3 times/week: 74 vs. 93	*p* = NS
Chin & Mohd Nasir 2009 [31]	Meal frequency (meals/daily)	Eating companions	Family vs. Peer vs. Alone (%) Never skip any meals: 38.7 vs. 33.3 vs. 12.9 Skipped at least one meal: 52.7 vs. 47.6 vs. 61.3 Skipped all three meals daily: 12.9 vs. 61.3 vs. 25.8	*p* < 0.05
		Ethnicity	Malay vs. Chinese vs. Indian (%) Never skip any meals: 27.1 vs. 51.3 vs. 57.7 Skipped at least one meal: 56.8 vs. 45.2 vs. 3.5 Skipped all three meals daily: 16.2 vs. 3.5 vs. 0	*p* < 0.05
		Living arrangement	Staying with family vs. In school hostel (%) Never skip any meals: 36.8 vs. 31.4 Skipped at least one meal: 53.4 vs. 48.6 Skipped all three meals daily: 9.8 vs. 20	*p* = 0.051
Nurul-Fadhilah et al. 2013 [37]	Snacking frequency (times/day)	Gender	Snacking frequency Male vs. Female (Mean ± SD) ♂ 1.86 ± 1.0 vs. ♀ 2.4 ± 1.1	*p* < 0.001
Rezali et al. 2015 [35]	Snacking frequency (days/week)		Male vs. Female (Mean ± SD)	
			Breakfast: ♂ 5.2 ± 2.1 vs. ♀ 4.7 ± 2.6	*p* < 0.05
			Lunch: ♂ 5.9 ± 1.8 vs. ♀ 5.8 ± 2.0	*p* = NS
			Dinner: ♂ 6.0 ± 1.9 vs. ♀5.8 ± 2.0	*p* = NS

Note: ♂, Male; ♀, Female; (3M + 3S), 3 meals + 3 snacks; (3M + 2S), 3 meals + 2 snacks; (3M + 1S), 3 meals + one snack; (3M), 3 meals; (≤2M ± 2,3S), meal skippers consumed snacks frequently; (≤2M ± 0,1S), meal skippers consumed snacks only one time or never; HEI, healthy eating index; SE, standard error; NS: Not Statistically Significant (*p* > 0.05); CI: Confidence interval; OR: Odd ratio.

**Table 3:** Summary of associations between physical activity determinants and physical activity behaviours.

Correlate	Author, Year [Ref]	Outcome	Association	*p*-Value
Demographics				
Age	Baharudin et al. 2014 [40]	Physical inactivity	Inactive vs. Active, OR (95% CI) 1.2 (1.16–1.23)	*p* < 0.001
	Cheah et al. 2016 [44]	PA	−0.075 (0.101)	*p* = NS
Gender	Baharudin et al. 2014 [40]	Physical inactivity	Female vs. Male (ref), OR (95% CI) 2.9 (2.66–3.10)	*p* < 0.001
	Aniza et al. 2009 [41]		Female vs. Male (ref), OR (95% CI) 2.176 (1.225–3.866)	*p* = 0.008
	Farah Wahida et al. 2011 [43]	PA level, MVPA	Male vs. Female: (%) Low: ♂ 65.0 vs. ♀ 82.7 Moderate: ♂ 35.0 vs. ♀ 17.3 High: ♂ 0 vs. ♀: 0	*p* < 0.001
	Dan et al. 2011 [42]	PA	Male, Beta: 2.366	*p* = 0.0001
	Cheah et al. 2016 [44]		Male vs. Female (ref): ♂ 0.603 (0.062)	*p* < 0.01
	Nurul-Fadhilah et al. 2013 [37]		Male vs. Female (Mean ± SD) ♂ 2.1 ± 1.7 vs. ♀ 1.3 ± 0.9	*p* < 0.001
	Su et al. 2014 [47]		Male vs. Female Mean (95% CI) ♀ 2.02 (1.91–2.12) vs. ♂ 2.46 (2.29–2.64)	*p*< 0.001
	Abdul Majid et al. 2016 [27]		Female Median (IQR)	
			Rural: 2.09 (1.72–2.43) in 2012	*p* = 0.006
			1.93 (1.56–2.28) in 2014	*p* = NS
	Cheah et al. 2012 [46]		Male vs. Female (Mean ± SD)	
			Before school: 26.1 ± 22.08 vs. 26.7 ± 23.71	*p* = NS
			During school: 37.7 ± 36.42 vs. 38.6 ± 36.70	*p* = NS
			After school: 47.4 ± 37.60 vs. 43.8 ± 35.62	*p* = NS
			Total time: 111.1 ± 77.70 vs. 109.1 ± 75.45	*p* = NS
	Teo et al. 2014 [38]		Male vs. Female (Median (95%, CI)	
			Malay ♂1.7 (1.8–2.4) vs. ♀1.1 (1.2–1.5)	*p* < 0.001
			Chinese ♂ 1.4 (1.6–2.4) vs. ♀ 0.8 (1.0–1.5)	*p* < 0.01
		MVPA duration (h/day)	Male vs. Female (Median (95%, CI)	
			Malay ♂1.3 (1.5–2.1) vs. ♀ 0.4 (0.5–0.8)	*p* < 0.001
			Chinese ♂ 1.0 (1.4–2.1) vs. ♀ 0.4 (0.6–1.0)	*p* < 0.001
Ethnicity	Aniza et al. 2009 [41]	PA	Inactive vs. Active (%) Malay 17.3 vs. 82.7; Others 27.3 vs. 72.7	*p* = 0.007
	Dan et al. 2011 [42]		Malay vs. Chinese (%) Low: 38.2 vs. 32.1 Moderate/High: 61.8 vs. 67.9	*p* = NS
	Su et al. 2014 [47]		Malay vs. Chinese vs. Indian vs. Others (Mean (95% CI) 2.21 (2.18–2.24) vs. 1.92 (1.72–2.17) vs. 2.31 (2.03–2.59) vs. 2.50 (2.31–2.68)	*p* < 0.05
	Cheah et al. 2016 [44]		Chinese & Indian/other vs. Malay(ref)	
			Chinese: −0.496 (0.086)	*p* < 0.01
			Indian/other: −0.042 (0.115)	*p* = NS
	Abdullah et al. 2016 [34]		Malay vs. Chinese (Mean ± SD) 2.8 ± 1.7 vs. 3.0 ± 2.3	*p* = NS
Maternal employment	Aniza et al. 2009 [41]	Physical inactivity	Not working vs. Working (ref), OR (95% CI) 2.167 (1.263–3.717)	*p* = 0.005
Paternal education	Dan et al. 2011 [42]	PA	r = 0.105	*p* < 0.05
	Cheah et al. 2016 [44]		Primary vs. Secondary vs. Tertiary(ref) −0.106 (0.131) vs. −0.052 (0.084)	*p* = NS, *p* = NS
Maternal education	Dan et al. 2011 [42]	PA	r = 0.08	*p* = NS
	Cheah et al. 2016 [44]		Primary vs. Secondary vs. Tertiary (ref) −0.248 (0.0130) vs. −0.293 (0.090)	*p* < 0.1, *p* < 0.01
Household income	Dan et al. 2011 [42]	PA	r = 0.08	*p* = NS
Household size			r = 0.03	*p* = NS
	Cheah et al. 2016 [44]		0.062 (0.016)	*p* < 0.01
Parent’s marital status	Cheah et al. 2016 [44]		Married vs. Divorced/widowed (ref) 0.059 (0.137)	*p* = NS
Physical-Environmental				
School session	Baharudin et al. 2014 [40]	PA	Noon vs. Morning (ref), OR (95% CI) 1.3 (1.13–1.44)	*p* < 0.001
Place of residence	Su et al. 2014 [47]		Rural vs. urban (Mean (95% CI) 2.14 (1.95–2.32) vs. 2.34 (2.25–2.43)	*p* = NS
	Abdul Majid et al. 2016 [27]		Rural (Median (IQR) Rural: 2.24 (1.90–2.70) in 2012 2.12 (1.70–2.64) in 2014	*p* = 0.013
Hot weather	Aniza et al. 2009 [41]	PA	Inactive vs. Active (%) Yes: 25.3 vs. 74.7 No: 20 vs. 80	*p* = 0.031
Equipment not available			Inactive vs. Active (%) Yes: 26.9 vs. 73.1 No: 20.7 vs. 79.3	*p* = 0.023
Facility far from home			Inactive vs. Active (%) Yes: 25.6 vs. 74.4 No: 19.7 vs. 80.3	*p* = 0.026
Traffic safety	Cheah et al. 2012 [46]		r = −0.15	*p* = NS
Residential density			r = 0.072	*p* = NS
Land-use mix diversity			r = 0.074	*p* = NS
Land-use mix access			r = 0.43	*p* = NS
Street connectivity			r = −0.03	*p* = NS
Infrastructure for walking			r = −0.078	*p* = NS
Aesthetics			r = −0.041	*p* = NS
Safety from crime			r = −0.046	*p* = NS
Neighborhood satisfaction			r = −0.009	*p* = NS
Facility support	Abd-Latif et al. 2012 [45]	PA involvement	r = 0.069	*p* = 0.038
Usage level of facilities			r = 0.094	*p* < 0.05
Safety			r = 0.002	*p* = NS
Social-Environmental				
Family without exercise	Aniza et al. 2009 [41]	PA	Inactive vs. Active (%) Yes: 27.1 vs. 72.9 No: 16.9 vs. 83.1	*p* = 0.005
Physical education	Cheah et al. 2016 [44]		0.151 (0.018)	*p* < 0.01
Social influence	Dan et al. 2011 [42]		Peer; Beta = 0.339	*p* = 0.0001
Family influence			r = 0.298	*p* < 0.001
Behavioral				
Breakfast intake	Baharudin et al. 2014 [40]	Physical inactivity	None, Irregular vs. Daily(ref), OR (95% CI)	
			1.9 (1.74, 2.13)	*p* < 0.001
			1.4 (1.33, 1.55)	*p* < 0.001
Stretching is important before exercise	Aniza et al. 2009 [41]	PA	No vs. Yes (ref), OR (95% CI) 3.747 (1.540–9.118)	*p* = 0.004
Time constraint			Yes vs. No (ref), OR (95% CI) 2.473 (1.335–4.579)	*p* = 0.004
Exercise when having ample time			No vs. Yes (ref) OR (95% CI) 2.482 (1.413–4.360)	*p* = 0.002
No skills to participate in PA			Inactive vs. Active (%) Yes: 27.3 vs. 72.7 No: 19.3 vs. 80.7	*p* = NS
Prefer to watch TV			Inactive vs. Active (%) Yes: 24.9 vs. 75.1 No: 9.1 vs. 90.9	*p* = 0.005
Embarrassed			Inactive vs. Active (%) Yes: 30 vs. 70 No: 21.4 vs. 78.6	*p* = 0.028
Being lazy			Inactive vs. Active (%) Yes: 30 vs. 70 No: 21.4 vs. 78.6	*p*=<0.0001
Too troublesome			Inactive vs. Active (%) Yes: 32.5 vs. 67.5 No: 20.6 vs. 79.4	*p* = 0.005

Note: ♂, Male; ♀, Female; SE, standard error; NS, Not Statistically Significant (*p* > 0.05); CI, Confidence interval; OR, Odd ratio; IQR, Interquartile Range; PA, Physical Activity.

## Supplementary Materials

The following is available online at http://www.mdpi.com/1660-4601/16/4/603/s1, Text S1: Search strategies; Text S2: Newcastle-Ottawa Scale for cross-sectional and cohort studies; Table S1: PRISMA checklist; Tables S2 and S3: Quality assessment of the studies.
