# Supplementary material for: Determinants of Diet and Physical Activity in Malaysian Adolescents: A Systematic Review
Source: Int J Environ Res Public Health. 2019 Feb 19;16(4):603. doi: 10.3390/ijerph16040603 (PMC6406561; doi:10.3390/ijerph16040603)
Supplement: Supplementary file 1 [file ijerph-16-00603-s001.zip › Table S2,S3. Quality assessment of studies.docx]

**Table S2.** Quality assessment of cross-sectional studies.

| **Author, Year**  **[Ref]** | **Representativeness of the Sample** | | **Sample Size** | | **Non-Respondents** | | **Ascertainment of the Exposure** | | **Comparability** | | **Assessment of the Outcome** | | **Statistical Test** | | **Total Score (Selection+**  **Comparability**  **+ Outcome)** | **Quality Score (Good/Fair/**  **Poor)** |
| --- | --- | --- | --- | --- | --- | --- | --- | --- | --- | --- | --- | --- | --- | --- | --- | --- |
|  | **Score** | **Reason for Score** | **Score** | **Reason for Score** | **Score** | **Reason for score** | **Score** | **Reason for Score** | **Score** | **Reason for score** | **Score** | **Reason for score** | **Score** | **Reason for Score** |  |  |
| Abdullah et al. 2016 [34] | b-1 | Community approaches  & peer-to-peer referral | a-1 | Sample size determined  based on outcome of bone health assessments | c | No referral to  non-respondents | a-2 | Validated  measurement tool | a-1,  b-1 | Adjusted for age, gender, ethnicity and SES + other dietary/PA factors | 0 | FFQ/ Self-report | a-1 | SE and  p-values are provided, tests are described | 4+2+1= 7 | Good |
| Rezali et al. 2015 [35] | a-1 | Schools randomly selected and all adolescents invited | b | No justification | c | No referral to  non-respondents | b-1 | Non-validated measurement tool | 0 | Study does not adjust for SES | c-1 | Self-report | a-1 | The probability level and tests are described | 2+0+2 | Poor |
| Majid et al. 2016 [48] | a-1 | Schools randomly selected and all adolescents invited | a-1 | Justified and satisfactory | c | No referral to  non-respondents | b-1 | Non-validated measurement tool | 0 | Study does not adjust for SES | c-1 | Self-report | a-1 | The probability level and tests are described | 3+0+2 | Poor |
| Loh et al.  2017 [36] | b-1 | Multi-stage sampling | b | No justification | c | No referral to  non-respondents | a-2 | Validated measurement tool | 0 | Study does not adjust for SES | c-1 | Self- report | b | The probability level are not described | 3+0+1=4 | Poor |
| Nurul-Fadhilah et al. 2013 [37] | b-1 | Convenience sample | b | No justification | c | No referral to  non-respondents | a-2 | validated measurement tool | 0 | Study does not adjust for SES | c-1 | Self-report | a-1 | The probability level and tests are described | 3+0+2=5 | Poor |
| Teo et al. 2014 [38] | b-1 | Not random sampling | b | No justification | c | No referral to  non-respondents | a-2 | Validated measurement tool | 0 | Study does not adjust for SES | c-1 | Self-report | a-1 | The probability level and tests are described | 3+0+2 | Poor |
| Boon et al.  2012 [39] | a-1 | Schools randomly selected and all adolescents invited | b | No justification | c | No referral to  non-respondents | b-1 | Non-validated measurement tool | 0 | Study does not adjust for SES | c-1 | Self-report | a-1 | The probability level and tests are described | 2+0+2=4 | Poor |
| Cynthia et al. 2013 [49] | a-1 | Schools randomly selected | a-1 | Calculated based on prevalence (70%) of children having family meals more than 3 times weekly | c | No referral to  non-respondents | b-1 | Non-validated measurement tool | 0 | Study does not adjust for SES | c-1 | Self-report | a-1 | The probability level and tests are described | 3+0+2 | Poor |

**Table S2.** *Cont.*

| **Author, Year**  **[Ref]** | **Representativeness of the Sample** | | **Sample Size** | | **Non-Respondents** | | **Ascertainment of the Exposure** | | **Comparability** | | **Assessment of the Outcome** | | **Statistical Test** | | **Total Score (Selection+**  **Comparability**  **+Outcome)** | | **Quality Score (Good/**  **Fair/Poor)** | |
| --- | --- | --- | --- | --- | --- | --- | --- | --- | --- | --- | --- | --- | --- | --- | --- | --- | --- | --- |
|  | **Score** | **Reason for Score** | **Score** | **Reason for Score** | **Score** | **Reason for Score** | **Score** | **Reason for Score** | **Score** | **Reason for Score** | **Score** | **Reason for Score** | **Score** | **Reason for Score** |  |  |  |  |
| Chin & Mohd Nasir 2009 [31] | a-1 | Schools randomly selected and all adolescents invited | b | No justification | c | No referral to  non-respondents | b-1 | Non-validated measurement tool | 0 | Study does not adjust for SES | c-1 | Self- report | a-1 | The probability level and tests are described | 2+0+2=4 | | Poor | |
| Baharudin et al. 2014 [40] | b-1 | Response rate based  on population level was 21% | a-1 | Selection based on probability  proportional to enrolment size | b | Characteristics of the whole population and non-respondents with calculation can figure out | a-2 | Validated  Measurement tool | 0 | Study does not adjust for SES | c-1 | Self-report | a-1 | The probability level and tests are described | 4+0+2=6 | | Poor | |
| Aniza et al. 2009 [41] | a-1 | Schools randomly selected | b | No justification | c | No referral to  non-respondents | b-1 | Non-validated measurement tool | 0 | Study does not adjust for SES | c-1 | Self-report | b | The probability level are not described | 2+0+1=-3 | Poor | |  |
| Dan et al.  2011 [42] | a-1 | Schools randomly selected and all adolescents invited | b | No justification | c | No referral to  non-respondents | b-1 | Non-validated measurement tool | 0 | Study does not adjust for SES | ac-1 | Self-report | a-1 | The probability level and tests are described | 2+0+2=4 | Poor | |  |
| Farah Wahida et al. 2011 [43] | a-1 | Schools randomly selected and all adolescents invited | a-1 | Justified and satisfactory | c | No description of the  non-respondents' characteristics | b-1 | Non-validated measurement tool | 0 | Study does not adjust for SES | c-1 | Self-report | a-1 | The probability level and tests are described | 3+0+2=5 | Poor | |  |
| Cheah et al. 2016 [44] | b-1 | Non-random sampling-response rate was 89.55% | b | No justification | a-1 | Description of the  non-respondents' characteristics | b-1 | Non-validated measurement tool | 0 | Study does not adjust for SES | c-1 | Self-report | a-1 | The probability level and tests are described | 3+0+2 =5 | Poor | |  |
| Abd-Latif  et al. 2012 [45] | a-1 | Cluster sampling or multistage sampling | b | No justification on why involves the sample from selected level | c | No referral to  non-respondents | b-1 | Non-validated measurement tool | 0 | Study does not adjust for SES | c-1 | Self-report | b | Tests are not described | 2+0+1=3 | Poor | |  |

**Table S2.** *Cont.*

| **Author, year**  **[ref]** | **Representativeness of the Sample** | | **Sample Size** | | **Non-Respondents** | | **Ascertainment of the Exposure** | | **Comparability** | | **Assessment of the Outcome** | | **Statistical Test** | | **Total score (Selection+**  **Comparability**  **+Outcome)** | **Quality Score (Good/Fair/Poor)** |
| --- | --- | --- | --- | --- | --- | --- | --- | --- | --- | --- | --- | --- | --- | --- | --- | --- |
|  | **Score** | **Reason for Score** | **Score** | **Reason for Score** | **Score** | **Reason for Score** | **Score** | **Reason for Score** | **Score** | **Reason**  **for Score** | **Score** | **Reason for Score** | **Score** | **Reason for Score** |  |  |
| Cheah et al.  2012 [46] | b-1 | Representative of the average in the target population/non random sampling | a-1 | Justified and satisfactory | c | No referral to  non-respondents | b-`1 | Non-validated measurement tool | 0 | Study does not adjust for SES | c-1 | Self-report | a-1 | The probability level and tests are described | 3+0+2=5 | Poor |
| Su et al.  2014 [47] | a-1 | Schools randomly selected and all adolescents invited | a-1 | Justified and satisfactory | c | No referral to  non-respondents | a-1 | Validated measurement tool | 0 | Study does not adjust for SES | c-1 | Self-report | a-1 | The probability level and tests are described | 3+0+2=5 | Poor |

**Note: Good quality,** 3 or 4 stars in selection domain AND 1 or 2 stars in comparability domain AND 2 or 3 stars in outcome/exposure domain ; **Fair quality,** 2 stars in selection domain AND 1 or 2 stars in comparability domain AND 2 or 3 stars in outcome/exposure domain ; **Poor quality**, 0 or 1 star in selection domain OR 0 stars in comparability domain OR 0 or 1 stars in outcome/exposure domain.

**Table S3.** Quality assessment of cohort study.

| **Author, Year**  **[Ref]** | **Representativeness of the Sample** | | **Selection of Non-Exposed Cohort** | | **Ascertainment of Exposure** | | **No Demonstration for Outcome of Interest** | | **Comparability** | | **Assessment of the Outcome and Follow-Up Length** | | **Adequacy of Follow-Up**  **of Cohorts** | | **Total score (Selection+**  **Comparability**  **+Outcome)** | **Quality Score (Good/Fair/**  **Poor)** |
| --- | --- | --- | --- | --- | --- | --- | --- | --- | --- | --- | --- | --- | --- | --- | --- | --- |
|  | **Score** | **Reason for Score** | **Score** | **Reason for Score** | **Score** | **Reason for Score** | **Score** | **Reason for Score** | **Score** | **Reason for Score** | **Score** | **Reason for Score** | **Score** | **Reason for score** |  |  |
| Majid et al.  2016 [27] | a-1 | Somewhat representative of exposed cohort | c | No description of the derivation of the non-exposed cohort | b-1 | Structured interview | a-1 | No outcome of interest | 0 | Study does not adjust for SES | a-1 a-1 | Yes Yes | a-1 | Subjects lost for follow-up less than or equal to 20% suggested no different from those followed | 3+0+3=6 | Poor |

***Note:* Good quality,** 3 or 4 stars in selection domain AND 1 or 2 stars in comparability domain AND 2 or 3 stars in outcome/exposure domain; **Fair quality,** 2 stars in selection domain AND 1 or 2 stars in comparability domain AND 2 or 3 stars in outcome/exposure domain; **Poor quality**, 0 or 1 star in selection domain OR 0 stars in comparability domain OR 0 or 1 stars in outcome/exposure domain.
