# Supplementary material for: Determinants of Diet and Physical Activity in Malaysian Adolescents: A Systematic Review
Source: Int J Environ Res Public Health. 2019 Feb 19;16(4):603. doi: 10.3390/ijerph16040603 (PMC6406561; doi:10.3390/ijerph16040603)
Supplement: Supplementary file 1 [file ijerph-16-00603-s001.zip › Text S2.Newcastle-Ottawa Scale.docx]

**Newcastle-Ottawa Scale adapted for cross-sectional studies**

**Selection: (Maximum 5 stars)**

**1) Representativeness of the sample:**

a) Truly representative of the average in the target population. * (all subjects or random sampling)

b) Somewhat representative of the average in the target population. * (non-random sampling)

c) Selected group of users.

d) No description of the sampling strategy.

**Note:** *Consider response rate in whether the random sampling was successful.*

**2) Sample size:**

a) Justified and satisfactory. *

b) Not justified.

**Note:** *Includes some kind of statement on how the sample size was decided upon (ideally based on a power calculation for a specific outcome).*

**3) Non-respondents:**

a) Comparability between respondents and non-respondents’ characteristics is established, and the response rate is satisfactory. *

b) The response rate is unsatisfactory, or the comparability between respondents and non-respondents is unsatisfactory.

c) No description of the response rate or the characteristics of the responders and the non-responders.

**Note:** *Define this; 40-50% response rate= unsatisfactory cut point.*

**4) Ascertainment of the exposure (risk factor):**

a) Validated measurement tool. **

b) Non-validated measurement tool, but the tool is available or described. *

c) No description of the measurement tool.

**Comparability: (Maximum 2 stars)**

***1) The subjects in different outcome groups are comparable, based on the study design or analysis.*** Confounding factors are controlled.

a) The study controls for the most important factor (select one). *

b) The study control for any additional factor. *

**Note:** *Age and gender and ethnicity and socioeconomic status should be adjusted for as a minimum to get this point. Only allocate this point if they have adjusted for the 4 variables above (option a).*

**Outcome: (Maximum 3 stars)**

**1) Assessment of the outcome:**

a) Independent blind assessment. **

b) Record linkage. **

c) Self report. *

d) No description.

**Note:** *Equivalent to objective measure of health as opposed to self-report*

*If the outcome is diet, then options Will change accordingly*

*a) Weighed record or direct observation***

*b) Multiple day recall, diet history or diary**

*c) Single day recall, or diary or FFQ (0 stars)*

**2) Statistical test:**

a) The statistical test used to analyze the data is clearly described and appropriate, and the measurement of the association is presented, including confidence intervals and the probability level (p value). *

b) The statistical test is not appropriate, not described or incomplete.

**Note:** *Or standard error or some measures of variability of association estimate.*

This scale has been adapted from the Newcastle-Ottawa Quality Assessment Scale for cross-sectional studies to perform a quality assessment of cross-sectional studies for the systematic review,

Thresholds for converting the Newcastle-Ottawa scales to AHRQ standards (good, fair, and poor):

**Good quality:** 3 or 4 stars in selection domain AND 1 or 2 stars in comparability domain AND 2 or 3 stars in outcome/exposure domain

**Fair quality:** 2 stars in selection domain AND 1 or 2 stars in comparability domain AND 2 or 3 stars in outcome/exposure domain

**Poor quality:** 0 or 1 star in selection domain OR 0 stars in comparability domain OR 0 or 1 stars in outcome/exposure domain.

**Newcastle-Ottawa Quality Assessment Form for Cohort Studies**

**Note:** A study can be given a maximum of one star for each numbered item within the Selection and Outcome categories. A maximum of two stars can be given for Comparability.

**Selection**

1) **Representativeness of the exposed cohort**

a) Truly representative (one star)

b) Somewhat representative (one star)

c) Selected group

d) No description of the derivation of the cohort

2) **Selection of the non-exposed cohort**

a) Drawn from the same community as the exposed cohort (one star)

b) Drawn from a different source

c) No description of the derivation of the non-exposed cohort

3) **Ascertainment of exposure**

a) Secure record (e.g., surgical record) (one star)

b) Structured interview (one star)

c) Written self-report

d) No description

e) Other

4) **Demonstration that outcome of interest was not present at start of study**

a) Yes (one star)

b) No

**Comparability**

1) Comparability of cohorts on the basis of the design or analysis controlled for confounders

a) The study controls for age, sex and marital status (one star)

b) Study controls for other factors (list) (one star)

c) Cohorts are not comparable on the basis of the design or analysis controlled for confounders

**Outcome**

**1) Assessment of outcome**

a) Independent blind assessment (one star)

b) Record linkage (one star)

c) Self report

d) No description

e) Other

**2) Was follow-up long enough for outcomes to occur**

a) Yes (one star)

b) No

Indicate the median duration of follow-up and a brief rationale for the assessment above:

**3) Adequacy of follow-up of cohorts**

a) Complete follow up- all subject accounted for (one star)

b) Subjects lost to follow up unlikely to introduce bias- number lost less than or equal to 20% or description of those lost suggested no different from those followed. (one star)

c) Follow up rate less than 80% and no description of those lost

d) No statement

Thresholds for converting the Newcastle-Ottawa scales to AHRQ standards (good, fair, and poor):

**Good quality:** 3 or 4 stars in selection domain AND 1 or 2 stars in comparability domain AND 2 or 3 stars in outcome/exposure domain

**Fair quality:** 2 stars in selection domain AND 1 or 2 stars in comparability domain AND 2 or 3 stars in outcome/exposure domain

**Poor quality:** 0 or 1 star in selection domain OR 0 stars in comparability domain OR 0 or 1 stars in outcome/exposure domain
